# Supplementary material for: Changes in the Epidemiology of Hepatocellular Carcinoma in Carinthia, Austria, 2012–2023
Source: Cancers (Basel). 2023 Oct 30;15(21):5215. doi: 10.3390/cancers15215215 (PMC10649038; doi:10.3390/cancers15215215)
Supplement: Supplementary file 1 [file cancers-15-05215-s001.zip › cancers-2598992-supplementary.pdf]

Supplementary Table S1. ALBI Score Grading for various BCLC Stages

		ALBI-Score			
		**Grade 1**	**Grade 2**	**Grade 3**	
		N (%)	N (%)	N (%)	P value
**BCLC**	1	46 (41.8)	59 (53.6)	5 (4.5)	
	2	18 (30.5)	34 (57.6)	7 (11.9)	<0.001
	3	19 (27.5)	45 (65.2)	5 (7.2)	
	4	0 (0)	10 (30.3)	23 (69.7)	

Abbreviations: ALBI Albumin-Bilirubin-Score; BCLC Barcelona Clinic Liver Cancer

Supplementary Table S2. Survival time of patients in time-period 1 vs. time-period 2 including OLT

			**Overall survival TP 1 (months)**			**Overall survival TP 2 (months)**		**P value**
**Variable**		**N=128**	Median	95% CI	**N=148**	Median	95% CI	**(log rank)**
Age	<70	42	14.3	8.6–20.0	62	20.7	10.8–30.7	
	≥70	86	24.2	19.5–28.8	86	19.8	15.0–24.6	0.176
Sex	male	109	22.4	17.0–27.7	127	20.1	15.6–24.7	
	female	19	12.4	4.3–20.4	21	18.1	11.4–24.7	0.841
Liver cirrhosis	Present	106	21.6	15.5–27.7	101	20.2	7.5–65.9	
	Absent	22	22.0	8.7–35.4	47	36.7	16.0–24.3	0.279
Etiology	Alcohol	69	18.9	11.5–26.3	70	18.0	13.8–22.3	
	Viral	28	27.0	9.2–44.8	27	53.3	19.4–87.2	
	NASH	15	26.2	4.8–47.7	28			
	Other	6	21.9	16.8–27.0	7	14.1	0.0–39.5	0.004
Ascites	Present	28	5.4	0.0–11.7	41	11.8	1.5–22.1	
	Absent	98	24.2	19.7–28.7	106	27.8	11.5–44.1	<0.001
Child-Pugh	No cirrhosis	22	22.0	8.7–35.4	47	36.7	7.5–65.9	
	A	55	28.7	19.8–37.6	61	27.0	12.8–41.3	
	B	38	15.2	9.3–21.0	27	11.8	0.9–22.7	
	C	13	1.8	0.0–3.6	13	5.6	0.0–13.7	<0.001
BCLC	A	53	33.1	11.2–55.1	59	52.8	20.1–85.5	
	B	32	24.2	12.1–36.2	29	27.0	6.5–47.6	
	C	23	10.4	5.9–15.0	46	10.0	6.1–13.9	
	D	20	1.8	0.9–2.6	14	1.9	0.0–6.1	<0.001
Focality	Unifocal	55	22.0	15.4–28.6	72	52.8	20.6–85.0	
	Multifocal	73	21.9	16.0–27.8	76	12.8	8.9–16.6	0.002
Up-to-seven	≤7	65	28.5	14.9–42.1	76	34.6	7.7–61.4	
	>7	62	12.9	8.5–17.2	72	11.0	8.3–13.8	<0.001
SBL	Present	120	22.2	18.0–26.4	16	10.5	5.7–15.2	
	Absent	7	2.2	1.7–2.7	132	21.2	12.5–29.9	<0.001
MVI	Present	105	24.2	19.6–28.7	36	4.8	3.2–6.4	
	Absent	22	7.4	2.1–12.7	112	30.6	16.5–44.6	<0.001

Ln. >2cm	Present	23	12.6	8.6–29.3	18	4.8	2.2–7.4	
	Absent	103	22.7	16.5–29.0	127	27.0	16.5–37.6	<0.001
CRP	Raised	58	12.1	7.2–16.9	62	11.2	7.7–65.7	
	Normal	67	33.1	20.1–46.2	85	36.7	7.0–15.3	<0.001
CAD	Present	13	19.4	4.1–34.6	21	16.0	15.9–24.4	
	Absent	112	21.9	16.6–27.1	127	20.1		0.454
CKD	Present	10	14.3	12.2–16.4	19	42.8	0.0–86.0	
	Absent	115	22.0	17.6–26.5	128	20.2	16.0–24.3	0.299
Hypertension	Present	80	24.0	17.5–30.5	87	18.1	12.9–23.3	
	Absent	45	18.2	9.6–26.9	61	21.2	8.9–33.4	0.866
DM	Present	38	18.9	8.7–29.1	50	20.5	9.0–31.9	
	Absent	90	22.2	15.4–29.0	98	18.7	13.3–24.1	0.610
BMI	<25	28	18.9	3.4–34.4	47	19.8	9.3–30.3	
	≥25	77	25.4	18.2–32.6	91	20.5	9.1–31.8	0.674
AFP	Raised (>7)	76	15.2	9.6–20.7	80	14.1	9.8–18.3	
	Normal	49	33.2	21.3–45.1	67	34.6	5.3–63.8	<0.001
ALBI score	Grade 1	18	33.2	9.8–56.6	65	34.6	26.4–42-7	
	Grade 2	81	22.0	15.7–28.4	67	15.8	11.5–20.1	
	Grade 3	24	7.6	0.0–19.0	16	3.9	0.8–6.9	<0.001
First-line therapy	Surgical/ OLT	21	63.2	36.0–90.4	21			
	RFA/ MWA	13	48.7	13.9–83.5	32	52.8	0.0–109.7	
	TACE	55	24.0	20.1–27.8	21	27.0	15.9–38.1	
	Systemic	16	12.6	8.7–16.4	59	11.8	8.8–14.8	
	None	23	2.1	1.3–2.8	15	1.9	0.4–3.4	<0.001
ASA	Present	24	24.2	9.0–39.4	35	53.3	0.0–110.2	
	Absent	104	21.6	17.2–26.0	113	18.1	14.2–21.9	0.248

Abbreviations: BCLC, Barcelona-Clinic Liver-Cancer Staging, CRP, C-reactive protein, BMI, body mass index, AFP, alpha-fetoprotein, ALBI, albumin-bilirubin score, SBL, sclerotic bone lesions, ASA, acetylsalicylic acid, NASH, non-alcoholic steatosis hepatis, MVI, microvascular invasion, Ln., lymphnode, CAD, coronary artery disease, CKD, chronic kidney disease, DM, diabetes mellitus
